# Iatrogenic pediatric unilateral vocal cord paralysis after cardiac surgery: a review

**DOI:** 10.3389/fped.2024.1460342

**Published:** 2024-09-03

**Authors:** Amy Callaghan, Hamdy El-Hakim, Andre Isaac

**Affiliations:** ^1^ Division of Pediatric Surgery, Department of Surgery, Stollery Children’s Hospital, University of Alberta, Edmonton, AB, Canada; ^2^ Division of Otolaryngology-Head and Neck Surgery, Department of Surgery, University of Alberta, Edmonton, AB, Canada

**Keywords:** pediatrics, otolaryngology, cardiac surgery, pediatric surgery, unilateral vocal cord paralysis, laryngeal reinnervation, vocal cord paralysis, laryngeal mobility disorders

## Abstract

Unilateral vocal cord paralysis (UVCP) is a growing area of research in pediatrics as it spans across many specialties including otolaryngology, cardiology, general surgery, respirology, and speech language pathology. Iatrogenic injury is the most common cause of UVCP, however there is a wide range of data reporting the prevalence, symptom burden, and best treatment practice for this condition. The literature included systematic reviews and meta-analyses, retrospective studies and limited prospective studies. Overall, the literature lacked consistency in the diagnosis, treatment, and long-term outcomes of patients with UVCP. Many articles conflated bilateral vocal cord paralysis (BVCP) with UVCP and had limited data on the natural history of the condition. There was no consensus on objective and subjective measurements to evaluate the condition or best indications for requiring surgical intervention. Thyroplasty, injection medialization (IM) and recurrent laryngeal nerve reinnervation (RLNR) were the reported surgical interventions used to treat UVCP, however there was limited data on short and long-term surgical outcomes in children. More research is needed to determine the true prevalence, natural history, indications for surgical intervention and long-term outcomes for pediatric patients with this condition.

## Introduction

Vocal cord paralysis (VCP), defined as the loss of vocal cord movement due to a lack of neural supply to the internal laryngeal muscles, is a growing research area in pediatrics. VCP is a type of laryngeal mobility disorder affecting the vocal cords, which are a primary component of the larynx. The larynx is responsible for lower airway protection, swallowing function and phonation. There are extrinsic and intrinsic muscles of the larynx, which assist in gross and fine movement of its structures. The external muscles of the larynx are innervated in part by the ansa cervicalis, a branch of cervical nerves C1–C4 (1). The internal muscles of the larynx are innervated by the superior and inferior (recurrent) laryngeal nerve, stemming from the vagus nerve. Damage to the recurrent laryngeal nerve limits neural supply to the internal laryngeal muscles, causing loss of movement of the vocal cords and resulting in VCP. VCP can be unilateral (one-sided) or bilateral (both sides); these conditions differ in their etiologies, prognoses, and management.

The most common etiology for unilateral vocal cord paralysis (UVCP) is iatrogenic injury to the recurrent laryngeal nerve (RLN) (2–23), likely due to the path of the RLN along the carotid sheath and its close relation to the aortic arch (2). Cardiothoracic surgery, specifically patent duct arteriosus (PDA) ligation, is the most common cause of iatrogenic UVCP (2–23). Other causes of iatrogenic UVCP include thyroid surgery, esophageal surgery and other thoracic procedures (3–10). Severity of UVCP symptoms vary; the most common indication for otolaryngological intervention is dysphonia, followed by aspiration, swallow dysfunction and some studies report airway symptoms such as stridor and work of breathing (3, 4, 9, 11, 12). Though these symptoms seem to be most commonly associated with UVCP, there are varying reports in the literature describing the diagnosis, prevalence, and symptom burden of this condition. The techniques and documentation used to confirm the presence of UVCP, the lack of clear definition of UVCP diagnosis, and the retrospective nature of many UVCP studies, limit the accuracy of true prevalence reported in pediatric patients with UVCP. Symptom burden is difficult to evaluate based on the literature, due to lack of consistent reporting, and heterogeneity in the methods used to evaluate this condition both objectively and subjectively.

Though the prevalence of this condition is under increasing scrutiny, it varies widely in the literature (3, 4, 11, 13), and a standardized management plan does not exist. The natural history of UVCP is not well understood, as most studies are retrospective in their evaluations, and often conflate bilateral vocal cord paralysis (BVCP) with UVCP (3, 4, 9, 14). Some studies report spontaneous recovery of UVCP, though the rate of this varies widely across studies (15–19). There are reports of initial recovery, then relapse of symptoms as the child ages due to muscular atrophy of the hemi-larynx (5), with limited evidence and heterogenous research methods. If spontaneous recovery is not present and symptoms continue to impact the quality of life of the patient, surgical intervention may be offered.

There are various surgical techniques offered to pediatric patients with UVCP; however, limited long-term evaluation of the outcomes of these procedures and heterogeneity of objective and subjective symptom evaluation across studies have resulted in uncertainty about which surgical intervention may be best for specific patients. Overall, surgical intervention may be warranted if spontaneous recovery is unlikely or fails; the most common modalities being injection medialization (IM), thyroplasty and recurrent laryngeal nerve reinnervation (RLNR) (6, 20).

Injection medialization involves the injection of a dissolvable material into the larynx resulting in medialization of the vocal cord. This is used to temporarily to relieve symptoms, as all injectable material will eventually reabsorb. Thyroplasty surgery involves placing an implant lateral to the vocal cord, medializing it to allow for sufficient closure from the contralateral cord. Laryngeal reinnervation uses the anastomosis of a branch of the ansa cervicalis or other nearby nerve to the recurrent laryngeal nerve which reinstates neural input and enhances the bulk and tone of the internal laryngeal muscles. This allows for strengthened glottic closure and has been reported to result in long-term resolution (21–25). Most reports of these surgical interventions are limited by low participant numbers and case reports, as well as limited pre and post operative documentation.

There is no consensus on the appropriate timing to offer these interventions, and there are limited data on objective short and long-term outcomes of patients who have been treated both surgically and non-surgically (4, 13, 26, 27). A standardized protocol for the diagnosis, treatment, and evaluation of symptom burden for UVCP is needed in the pediatric population.

## Anatomy, physiology and embryology

1. Overview of laryngeal embryology
2. Overview of laryngeal anatomy
3. Overview of laryngeal nerves

### Laryngeal embryology

The larynx first develops in the embryonic stage of gestation and the fetus gains swallow function around 13 weeks of gestation. The nerves that innervate the larynx are derived from the fourth and sixth branchial arches, which also form the aortic arch and subclavian artery. During gestational development, the artery branching from the right side of the sixth branchial arch is obliterated, while the left side becomes the ductus arteriosus; this is important because the left laryngeal nerve develops a longer and closely related pathway with the left ductus arteriosus due to this difference in development.

There are also differences between the infant and adult larynx that must be considered in pediatric otolaryngologic surgery. The infant larynx is located more superiorly, consists of a higher proportion of cartilaginous tissue and is smaller in size than an adult larynx (28). Understanding the anatomic landmarks, function of laryngeal muscles, and developmental changes with age help surgeons provide best options and care for patients.

### Laryngeal anatomy

The extrinsic muscles of the larynx comprise of the infrahyoid (strap) muscles, which participate in moving the larynx and facilitate swallowing. The intrinsic muscles of the larynx are involved in vocal cord adduction and abduction by altering the position of the arytenoid, cricoid and thyroid cartilages. The posterior cricoarytenoid (PCA) muscle is the only intrinsic laryngeal muscle responsible for abduction of the vocal cords; together, the intrinsic laryngeal muscles coordinate respiration, swallowing and phonation.

### Laryngeal nerves

The ansa cervicalis is the primary nerve responsible for innervating extrinsic laryngeal muscles and if often involved in laryngeal reinnervation surgery. There is a superior branch and an inferior branch which traverse separate paths and join to form the ansa cervicalis. The superior branch descends along the carotid sheath and creates a loop with the inferior root of the ansa cervicalis. The inferior branch innervates the strap muscles and is often used in laryngeal reinnervation surgery due to its proximity to other nerves and innervation of extrinsic laryngeal muscles.

The superior laryngeal nerve (SLN) and inferior (recurrent) laryngeal nerve (RLN) stem from the vagus nerve and innervate the intrinsic muscles of the larynx. The SLN separates into an external branch and an internal branch. The external branch is primarily a motor nerve, responsible for innervating one internal laryngeal muscle, the cricothyroid muscle, which adducts the vocal cords. The internal branch of the SLN primarily serves as a sensory nerve, innervating the root of the tongue, piriform sinus and epiglottis.

**Figure 1:**
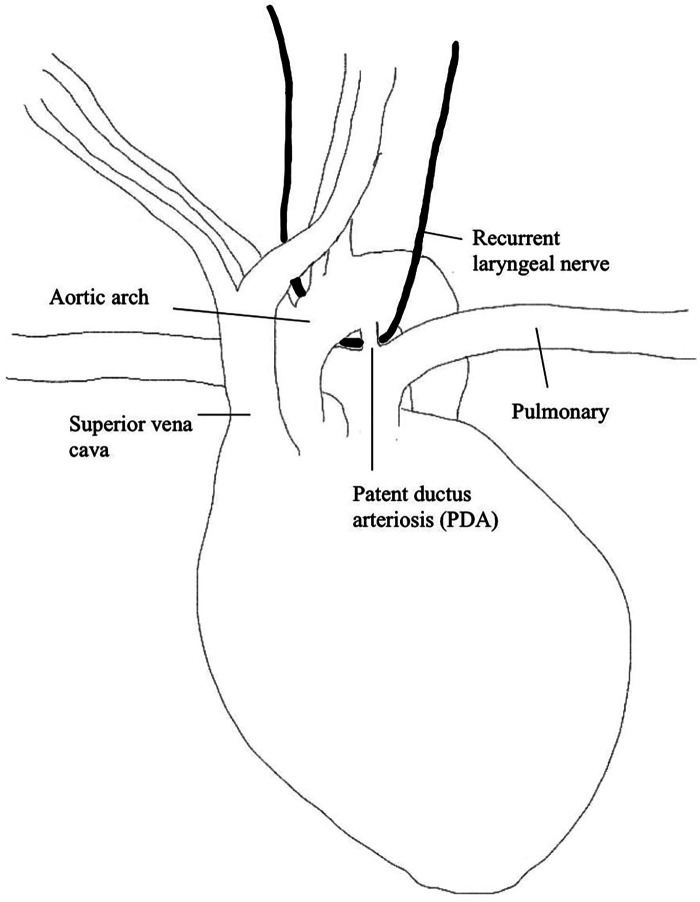
Anatomy of PDA and recurrent laryngeal nerve.

The right and left RLN differ in their anatomical pathways. The left RLN originates from the vagus nerve on the medial side of the inferior jugular vein, extends inferiorly along the carotid artery and crosses the aorta anteriorly, then loops around medially and extends upwards along the tracheoesophageal grove to the PCA (29). Since the left RLN extends around the aortic arch, it is prone to iatrogenic injury, specifically during cardiothoracic surgery (please refer to Figure 1). The right RLN loops around the subclavian artery rather than the aorta and inserts into the right PCA muscle. Both the left and right RLN enter the larynx posteriorly and have an anterior and posterior branch. The posterior branch innervates PCA while the anterior branch innervates the thyroarytenoid (TA) muscle and other intrinsic laryngeal muscles responsible for adducting the vocal cords.

## Laryngeal mobility disorders

1. Outline laryngeal mobility disorders
2. Describe definition, etiologies, and symptom measurement of UVCP
3. Describe UVCP diagnosis techniques

Though laryngeal paralysis is the main point of discussion, there are other laryngeal mobility disorders that may impact the movement and function of the larynx. Other examples of laryngeal mobility disorders include laryngeal dyskinesia, arytenoid dislocation, and arytenoid fixation. These disorders may present with similar symptoms to laryngeal paralysis; however, their treatment, management and prognosis may differ. When a patient presents with symptoms suggestive of laryngeal paralysis, it is important for physicians to conduct the appropriate examinations before concluding their diagnosis.

### Laryngeal paralysis

Laryngeal paralysis can be described as the interruption of the neural supply to the vocal cords, affecting their basic movement and/or sensory supply (3). This can cause airway, voice and swallowing difficulties, based on the severity and laterality of the condition; there are different diagnoses, prognoses and symptoms associated with paralysis in one or both cords. Bilateral vocal cord paralysis (BVCP) involves paralysis of both vocal cords, while unilateral vocal cord paralysis (UVCP) involves paralysis of one vocal cord (14). BVCP and UVCP are often conflated in the literature, however their etiologies, symptoms, treatments and prognoses can vary greatly (3, 4, 9, 14) (summarized in Table 1).

**Table 1:** Differences between UVCP and BVCP.

Vocal cord paralysis	Most common etiology	Common symptoms	Prognosis
Unilateral	Iatrogenic	• Hoarse/weak voice or cry	Unlikely to spontaneously recover (symptomatic <1 year)
		• Swallowing dysfunction	
		• Airway concerns	
Bilateral	Idiopathic	• Cyanosis	• More likely to have concomitant airway disease
		• Stridor/increased work of breathing	• More likely to require tracheostomy
		• Obstructive sleep apnea	• Higher rate of death
		• Swallowing dysfunction/feeding difficulties	

Most of the literature surrounding the natural history of laryngeal paralysis is retrospective in nature (3, 4, 9, 14). Cohen et al., who conducted a retrospective study on patients with laryngeal paralysis in 1982, found laryngeal paralysis was most commonly caused by idiopathic origin, followed by central nervous system (CNS) disease and surgical or blunt trauma accounting for only 11% of patients (14). This aligned with the literature at the time, however iatrogenic laryngeal paralysis has become more recognized and reported in the more recent literature. Two frequently referenced retrospective studies describing the natural history of vocal cord paralysis were published by Jabbour et al. (4) and Daya et al. (3). These reviews found iatrogenic injury to be the most common etiology of pediatric vocal cord paralysis. This may be due to higher survival rates of complex cardiac patients, evolving pediatric cardiac surgery techniques, increased awareness of UVCP, and the appearance of long-term complications. There is also evidence of a difference in etiologies of BVCP and UVCP in children compared to adults (9).

Aside from etiology of these conditions, the time of diagnosis varies between BVCP and UVCP. Jabbour et al. found almost 78.5% of patients were diagnosed with either UVCP or BVCP before 12 months of age (4). Rosin et al. reported that patients with BVCP are often diagnosed sooner, show symptoms sooner, require tracheostomies more often and have a higher rate of death than UVCP (9). Similar to Rosin's findings, Jabbour's study also found that BVCP patients were more likely to present with airway symptoms, have concomitant airway disease, and require tracheostomy (4, 9).

Studies on BVCP more often report airway or respiratory symptoms including cyanosis, stridor, and apneas (4, 9) whereas UVCP more commonly report dysphonia, including hoarseness or voice concerns reported, which is consistent across the literature (4, 9). BVCP and UVCP have also been noted to present feeding concerns (3). When evaluating the natural history of the condition, Jabbour found about half of the patients with BVCP or UVCP showed resolution of symptoms in the 2-year median follow up time, whether it was clinical resolution of symptoms or actual resolution of vocal cord function (4). About 25% of patients had resolution of symptoms with left UVCP, which is similar to other studies (17). While much of the literature conflates BVCP and UVCP, it is important to recognize the differences in prognoses and treatment of these conditions.

### Definition of UVCP

The nomenclature used to describe UVCP varies widely in the literature; the vocal cords may be referred to as vocal cords or vocal folds, and paralysis may often be used synonymously with paresis, palsy, or immobility, with lack of a consensus on the definition or differences in each. The misuse or lack of definition of this condition serves as a limitation to the literature around this topic; it leads to vague descriptions of the condition, risk of missed information due to heterogeneous vocabulary used when searching for studies, and lowered strength of further research due to lack of replicability and certainty in reported findings. There is also a lack of diagnostic confirmation reported in the literature. Most studies report UVCP based on flexible laryngoscopy or clinical symptoms, however this may lead to skewed numbers, as a flexible laryngoscopy cannot differentiate between a truly paralyzed vocal cord from fixation, paresis or dislocation of the arytenoid. This review will refer to the condition of unilateral vocal cord paralysis (UVCP) for consistency, however it is important to note there are varied terminologies used to describe this condition.

Along with the lack of consistency in vocabulary used to describe UVCP, there is a lack of definition of protocol for treatment and prognosis of this condition. Otolaryngological consultations for this patient population are most often requested due to dysphonia, feeding concerns, aspiration, stridor or work breathing (3, 11, 12, 17). The number of patients being diagnosed with VCP has increased in recent years; this may be due to advances in technology, but some research also suggests it can be due to the higher survival rate of neonates, who account for a large proportion of patients with VCP (30). Though the rate of diagnosis is increasing, there is insufficient data describing the prevalence of this condition or standardized protocols to diagnose and assess for UVCP symptoms in children.

### Causes

UVCP is usually diagnosed at a young age (under age 5) (31). The literature is consistent in reporting the high percentage of UVCP patients from cardiac surgeries (3, 4, 11, 13, 32), however a retrospective study that examined BVCP and UVCP in neonates noted several etiologies other than cardiac surgery (33). They discussed the percentage of UVCP patients that had idiopathic UVCP (37%), UVCP from birth complications (21%), neurological disorders (25%), or other etiologies such as cardiac or vascular malformations (17%) (33).

### Iatrogenic

The most common cause of UVCP is iatrogenic injury, specifically following cardiac procedures, the most common of which being patent duct arteriosus (PDA) ligation (4, 11, 13, 32, 34). Henry et al. compared PDA ligation surgical techniques, finding that a clipping technique rather than ligation technique led to less patients diagnosed with UVCP (16).

A common topic in the literature regarding UVCP diagnosis after surgery is the inconsistency in the diagnosis and threshold for investigating patients with vocal cord paralysis. Engeseth et al. published a systematic review on premature infants who underwent PDA ligation to evaluate the prevalence of UVCP and outcomes in this population (32). This study replicated similar findings to other highly cited studies, reporting that authors who systematically evaluated all patients via laryngoscopy after PDA ligation or heart surgery had a higher rate of UVCP than those who were only evaluated after presenting with symptoms (13, 16, 32). Orzell et al. found similar results to these studies, showing a large variance of UVCP diagnoses between <10% and >50% including articles reporting on post-operative outcomes of procedures other than PDA ligation, such as Fontan procedures, aortic arch repairs, and other cardiac procedures (15). This study found that the lowest range diagnosis was from a study that was symptom-based in their protocols, whereas the >50% statistic was based on a study that completed routine or universal screening for all patients post-cardiac surgery. Engeseth's study highlighted the discrepancies in diagnosis and systematic evaluation of patients undergoing heart surgery with regards to their vocal cord function and suggested having further systematic research done to identify the correct prevalence of this condition and reasonable treatment/diagnosis options (32).

Iatrogenic UVCP has been reported after other surgeries such as tracheoesophageal fistula repairs and thyroidectomy (35). More recent studies have shown that this complication after thyroid surgery has greatly diminished due to increased knowledge and understanding of the laryngeal nerve anatomy in relation to the thyroid gland, improved surgical techniques with higher volume surgeons and the implementation of nerve monitoring and other technological advances (2).

The most recent literature supports that iatrogenic injuries are the most common cause of UVCP in pediatric patients (4, 11, 13, 32, 34). This is becoming increasingly recognized in the literature, specifically pertaining to cardiothoracic surgery. This information helps surgeons and patients understand the risks of surgery; however, a systematic approach to post operative monitoring and assessment of UVCP is needed to allow of earlier diagnosis and treatment of this condition in the pediatric population.

### Idiopathic

Idiopathic VCP is more often found in BVCP than UVCP (4, 14, 32). Idiopathic UVCP in children has been reported to have a relatively high rate of recovery compared to other etiologies (32, 34).

### Birth trauma/neurological/prolonged intubation (4)

Birth trauma or complications resulting in c-sections or usage of forceps during delivery has been documented as an etiology for UVCP. One study reported birth trauma accounted for 20% of patients with laryngeal paralysis (14). Jabbour et al. conducted a systematic review on bilateral and unilateral VCP and found UVCP patients were more likely to have birth trauma etiologies than BVCP patients (7). Birth trauma UVCP was hypothesized to be most likely due to breach, forceps and vacuum-assisted deliveries (7).

Low birth weight has also been associated with increased risk for UVCP after cardiac surgery. This is thought to be due to the smaller size of the infant and organs, leading to a higher risk of injury to structures surrounding the aortic arch during cardiac surgery (16, 19). Multiple studies have reported the greater risk for UVCP in preterm infants or those with a lower birthweight (4, 16, 19).

CNS diseases such as cerebral palsy, Möbius’ syndrome, Arnold-Chiari malformation, and hydrocephalus may affect laryngeal function, however these etiologies are more commonly reported in BVCP cases than UVCP in children. UVCP from CNS disease is rare, stemming from conditions such as hypotonia or peripheral neurological diseases such as Horner syndrome (3, 33) and has been reported to have equally prevalent laterality (3, 14). The rate of recovery for UVCP from CNS is conflicting in the literature, where some studies suggest lower recovery rates with regards to swallowing function in CNS patients compared to iatrogenic UVCP (36) and other studies suggest UVCP from CNS disease has the highest rate of recovery. De Gaudemar et al. suggested there may be an association between recovery rates of UVCP from CNS disorders and birth trauma injuries, however this has not been examined or reported elsewhere.

Prolonged intubation has been reported to contribute to vocal cord paralysis (37). Zur et al. conducted a retrospective study measuring voice outcomes of patients who underwent phonosurgery for vocal cord paralysis (37). They found patients with a history of prolonged or repeated intubation had poorer voice outcomes post operatively due to the residual posterior glottic defect (Benjamin Defect) due to the placement of the endotracheal tube while intubated.

In earlier literature, idiopathic UVCP was thought to be the most common etiology of the condition, followed by CNS defects and birth trauma (33). More recently, iatrogenic UVCP, specifically after cardiothoracic surgery has been shown to be the largest contributor to the UVCP patient population in children. Though there are anatomical factors that put the recurrent laryngeal nerve at risk during these surgeries, awareness and diligence in the documentation and understanding of symptoms to diagnose UVCP in patients post operatively is vital in the treatment and prognosis of patients with this condition.

### Measurements of UVCP

Though many authors report on symptoms of UVCP, there is no consensus on standardized evaluations or measurements to indicate further investigations for patients presenting with this disease (14). Severity of symptoms and prognosis depend on the etiology, position of vocal cords, and compensatory movement of the contralateral vocal cord. The exact positioning of a paralyzed vocal cord may vary; Cohen et al. found about half of the patients with vocal cord paralysis had the vocal cords in the median position, though there are instances where the vocal cords can be immobile and lay in the paramedian or complete midline (14).

There are subjective and objective evaluations that can be used to document UVCP symptoms, however these reports are highly varied across UVCP literature. The most common symptom of UVCP and most common indication for otolaryngology referral is dysphonia; this is from the discretion of the providing care team and parent perception. Dysphagia, airway symptoms and other voice symptoms are also reported, but again do not have standardized testing to indicate need for referral to an otolaryngologist.

### Subjective

Common tools to assess voice quality that will be discussed are the Pediatric Voice-Related Quality of Life Survey (PVRQOL) (38), Pediatric Voice Handicap Index (pVHI) (39), Consensus Auditory-Perceptual Evaluation of Voice (CAPE-V) (40) testing and the Grade, roughness, breathiness, asthenia, and strain (GRBAS) scale (41) (please refer to Table 2). These measurements are subjective in nature, relying on parent or clinician perception to evaluate the symptom burden of UVCP and provide a numeric value to voice characteristics for comparison over time. Although they may be useful in determining the perceptual impact on the quality of life of the patient, the main limitation of these evaluation is the underlying bias of the patient, parent or clinician, as well as the variance in perception between observers.

**Table 2:** Subjective surveys for UVCP.

Name	Concern	Grading	Classification
PediEat	Swallowing	• Likert Scale	Normal, >1 SD from normal, >2 SD from normal
		• 0 (never)–5 (always)	
Infant and Toddler Swallowing Questionnaire	Swallowing	• Likert Scale	
		• Never—All the time	
CAPE-V	Voice	VAS rated 0–100 overall severity, roughness, breathiness, strain, pitch, loudness	• Mildly deviant
			• Moderately deviant
			• Severely deviant
			• Consistent
			• Intermittent
PVRQOL	Voice	• Likert scale	• Excellent
		• 0 (not a problem)–6 (not applicable)	• Fair to good
			• Poor to fair
			• Poor
			• Worst possible
pVHI	Voice	• Likert Scale	• (From adult VHI) • Low handicap
		• 0 (never)–4 (always)	• Moderate handicap
			• Severe handicap
GRBAS Scale	Voice	0 (no abnormalities)–3 (severe abnormalities)	• Normal • Mild Degree
			• Moderate degree
			• High degree

SD, standard deviation; CAPE-V, consensus auditory perceptual evaluation of voice; VAS, visual analog scale; PVRQOL, pediatric voice-related quality of life; pVHI, pediatric voice handicap index; GRBAS, grade, roughness, breathiness, asthenia, strain.

Subjective swallow evaluations reported in the literature are often based on patient or parent perception. This includes coughing and choking with feeding or reported aspiration symptoms. There are validated dysphagia questionnaires such as the PediEAT questionnaire (42) and the Infant and Toddler Swallowing Questionnaire (43), though there is little consistency across the literature in their utility (Table 2).

While some of the symptoms associated with UVCP are more obvious in their effect on patient quality of life, there is also evidence of psychological and social impacts including increased anxiety, anger, sadness, frustration, and poor communication/social skills for children with dysphonia (44–46). The influence these factors have on patient quality of life are rarely reported in the pediatric literature on UVCP.

Although there are validated measurements to assess voice and swallow symptoms in patients, there are varied uses of these in the literature, creating inconsistent findings and resulting in difficulty concluding true symptoms, outcomes, and best treatment practices for this condition.

### Objective

Objective evaluations of UVCP symptom burden include the analysis of voice recordings and the use of instrumental assessments to evaluate airway and swallow function. Acoustic analysis of voice is done by comparing acoustic properties such as maximum phonation time (MPT), shimmer, jitter and intensity of the voice to objectively evaluate voice function. This testing evaluates voice attributes through analytic software such as the Analysis of Dysphonia in Speech and Voice (ADSV™ Pentax Medical, New Jersey, USA) program. Objective measures are helpful in recording the qualitative values in voice, cough strength and overall function of the vocal cords or laryngeal structures. The main limitation of these measures is the difficulty to obtain consent and follow up from patients, as well as lack of equipment to analyze the collected data.

Instrumental assessments including flexible nasolaryngoscopy, rigid bronchoscopy, functional endoscopic evaluation of swallowing and video fluoroscopic swallow studies are used to objectively evaluate swallow and airway symptoms. These interventions allow for visualization of vocal cords to assess movement, function, and improvement over time. Though some of these tools are commonly used to assess and diagnose UVCP, there is a lack of documentation across studies and follow up protocols to objectively compare these evaluations over time.

A standardized method to subjectively and objectively diagnose, measure and evaluate UVCP symptom burden in pediatric patients would be highly useful in the diagnosis and management of these patients.

## Diagnosis of unilateral vocal cord paralysis

One weakness in the literature is the inconsistency in the approach to diagnosis of UVCP. There are also inconsistencies or lack of description of the vocal fold assessment, measures taken to confirm paralysis, and definition of resolution, whether it is clinical resolution (resolution of symptoms) or true resolution (mobility of vocal cords). For example, of the systematic reviews published on the natural history of vocal cord paralysis, only one study documented the palpation of the arytenoids to ensure the immobility was related to nerve function and not joint fixation (3), and one study reported the use of flexible laryngoscopy to analyze the resolution of symptoms (4); the lack of consistencies and lack of replicability serve as limitations in the literature regarding the diagnosis of VCP.

A consensus on the definition of these terms and systematic protocol to evaluate and diagnose unilateral vocal cord paralysis has yet to be established and is needed to sufficiently evaluate the prevalence of this condition.

### Flexible nasal laryngoscopy/suspension laryngoscopy

Flexible nasal laryngoscopy (FNL) is considered the reference standard test to assess the vocal cords of awake patients (47). One of the main advantages of this procedure is the ability to have the awake patient phonate while the camera is capturing the airway to visualize the movement or immobility of the vocal cords. The main disadvantage for this procedure in infants and children is the difficultly compared to adults due to poorer compliance, excess movement, secretions, and smaller anatomy. Commonly cited studies on UVCP natural history such as Jabbour, De Gaudemar and Truong use UVCP diagnosed by FNL as part of their inclusion criteria for UVCP diagnosis (4, 17, 33). This criteria is not consistently specified in surgical outcome reports such as those published by Aires and Butskiy (6, 20). FNL is often used to distinguish between “true” UVCP resolution where vocal cord movement has recovered and “clinical” UVCP resolution defined as parent or patient perceived resolution of symptoms (4).

FNL can also be done while the patient is feeding to assess for potential aspiration or swallowing dysfunction through a functional endoscopic evaluation of swallowing (FEES) assessment. Advantages of this test include the ability to visualize the airway while the patient is feeding and awake, ability to test different thicknesses and consistencies of food, and it does not expose the patient to any anesthetics or radiation. Disadvantages include the difficulty in younger patients to cooperate with the task, and limited view of the glottic structures at the peak of the swallow as the muscles contract and obstruct the camera visualization.

If the patient is unable to undergo a FEES assessment or has inconclusive findings, a video fluoroscopic swallow study (VFSS) may be warranted. A VFSS is normally completed by a speech language pathologist (SLP) in conjunction with a radiologist. This allows for full but indirect visualization of laryngeal structures during the swallow; however, it does subject the patient to radiation as it uses x-ray fluoroscopy to capture a video of the patient while swallowing.

The systematic use of swallow assessments for UVCP patients is not currently used, however there are more studies using swallowing data to evaluate UVCP surgical interventions on swallowing function (25, 48). Zur et al. noted the effect of RLNR on swallowing function through VFSS (25), and there is a handful of studies documenting instrumental swallow assessments before and after injection laryngoplasty (48, 49). Most recently, Sheen et al. published a study using VFSS to evaluate the effect of IM procedures on UVCP patients after cardiac surgery who were aspirating thin liquids. This is one of the few publications in UVCP literature using swallowing data with a control group (50).

Another option to visualize the upper airway is through a rigid or suspension laryngoscopy and bronchoscopy. This is often the examination that can definitively diagnose UVCP in patients, as it allows the surgeon to palpate the vocal cords and rule out any other causes of immobility, whether they are structural, or non-nerve related. Daya et al. was one of the only papers to specifically note the use of laryngoscopy to definitively diagnose UVCP in their patient pool (3). Rigid bronchoscopy can also be combined with electromyography (EMG) which tests nerve activity in the internal laryngeal muscles, which can assist in diagnosing UVCP. Disadvantages to this procedure include the risk of anesthetic, especially in the pediatric population, and inability for the patient to phonate or voluntarily move their vocal cords as with an FNL.

### Laryngeal electromyography

Laryngeal electromyography (EMG) is a tool used to measure motor unit action potentials in a muscle to determine if there is abnormal neurological activity (51). This assessment is normally done for children in the operating room under anesthetic with spontaneous breathing due to the invasive nature of EMG, which is a limitation of this test (31). Monopolar EMG is used to determine the PCA (responsible for abduction of the vocal cords) and TA (responsible for adducting the vocal cords) neural input and evaluate the synchronicity of action potentials with respiration rate. The utility of EMG recordings to diagnose and manage pediatric patients with UVCP is still under examination.

Koch et al. evaluated VCP in children and were the first to use monopolar laryngeal EMG as a diagnostic tool to measure the amount of neural activity to the PCA muscles (52). This technique has become more common in the diagnosis and treatment of UVCP patients. A systematic review on laryngeal reinnervation for pediatric UVCP by Hoey et al. reported 8/19 studies (42%) from their analysis had documentation of EMG recording pre-operatively, however there was limited and inconsistent documentation of EMG techniques in the literature (26). Maturo et al. used EMG on UVCP patients and found patients had a poor prognosis if there was no electrical signal in the TA or PCA after 6 months from the time of injury (53). They also found patients with UVCP after PDA ligation had worse prognoses than other etiologies.

While some studies show relevant data through laryngeal EMG for UVCP, this tool is limited due to the presence of noise between probes due to the monopolar technique, lack of consistency in TA and PCA testing, inconsistency in grading the severity of nerve damage, and technical challenges of the pediatric anatomy.

### Ultrasound

Ultrasound (US) has recently been used as a less invasive tool to aid in the diagnosis of VCP. This method is attractive in the pediatric space as it is not as invasive as a laryngoscopy and does not expose the child to radiation. This is also a more familiar modality for parents making it more comfortable and acceptable (10).

Horner et al. examined patients that underwent Norwood and aortic arch procedures to determine the prevalence of vocal cord immobility post operatively using US as a standardized initial test (54). US results lead to a 96% positive diagnosis rate when compared with FNL. US was performed three days after extubation to determine if otolaryngology consult was warranted. They found that 62% of patients showed vocal cord paralysis/paresis compared to the 32% in the pre-intervention group (54).

As laryngeal ultrasound is a new technique for the assessment of vocal cord movement, there is limited literature describing its utility in UVCP diagnosis. Some advocate US as a pre-diagnostic tool before proceeding to FNL or suspension laryngoscopy; however, training to operate and interpret the ultrasound is required, as well as the availability of the modality in that centre.

In summary, flexible nasal laryngoscopy and suspension laryngoscopy are the most common modalities used to assess vocal cord motion and diagnose UVCP in children. EMG and US can aid in the diagnosis and inform prognosis, however there are no standardized protocols in place to incorporate these techniques in the diagnosis workup of UVCP currently.

More research and discussion around the utility of assessment tools such as EMG and US are needed to create a systematic diagnostic protocol for UVCP in pediatric patients. This, along with standardization of symptom assessment tools and awareness of common etiologies for this condition would aid in determining the true prevalence and burden of symptoms in this patient population.

## Treatment options

1. Summarize current literature on natural history of UVCP
2. Describe literature on non-surgical treatment (spontaneous resolution)
3. Describe literature on surgical treatment

### Spontaneous resolution

There is a wide range of data evaluating the resolution of UVCP in patients (4, 11, 16, 17). Overall, iatrogenic vocal cord paralysis has been shown to have a lower rate of recovery than other etiologies (3, 11). and premature or low birth weight patients have been reported to have a lower rate of spontaneous resolution (16). Orzell et al. completed a systematic review evaluating swallowing and respiratory symptoms of patients with UVCP after congenital heart surgery (15). This study found swallow symptoms were relatively high, however full recovery of UVCP ranged from 8%–96%, exemplifying the wide range of reported resolution of this condition. They also noted a major flaw in the literature is a lack of FNL or suspension laryngoscopy to confirm vocal cord recovery; they reported studies do not consistently document the use of clinical assessments to confirm their diagnoses or resolution of symptoms.

These limitations persist across the literature of UVCP; the definition and recommended time frame to allow for spontaneous resolution are inconsistent and attrition of patients due to incomplete follow up result in widely varied statistics to describe the prevalence and resolution of UVCP. There are studies that suggest symptoms can resolve due to compensatory actions from the uninjured vocal cord (14), and define spontaneous resolution as the resolution of UVCP symptoms; other studies define spontaneous resolution clinically, with the regaining of vocal cord movement on endoscopic evaluation confirming spontaneous resolution of UVCP. The variance in description of spontaneous resolution, along with the heterogeneity of follow up data and recommendations to evaluate spontaneous resolution are prominent limitations in the literature around the natural history of UVCP.

Analyzing spontaneous recovery in UVCP, Biot et al. evaluated long term outcomes of patients who had cardiac surgery in a prospective observational study (18). This study gathered retrospective data from patients who underwent cardiac surgery between the years of 2010 and 2015. Patients were then contacted in 2011 with at least a 5-year time span from their cardiac surgery to complete the PediEAT survey to evaluate swallowing symptoms and the voice handicap index VHI to evaluate voice symptoms. Willing patients were also assessed by an otolaryngologist by flexible nasal laryngoscopy to determine if there was vocal cord paralysis. 3% of patients had documented vocal cord immobility after cardiac surgery and were followed up by the study team. Of those willing to participate in the study, 65% showed spontaneous recovery of vocal cord movement.

This data concurs with similar studies reporting that the recovery of vocal cord function is unlikely to occur longer than 1 year after surgery (17, 33). Quality of life questionnaires showed no difference in swallowing function between paralysis and mobile patients, but there was a significant difference between groups in the VHI quality of life score.

A major limitation of this study is the retrospective method to identify patients with laryngeal immobility. There is a lack of definition of laryngeal immobility, and a wide margin for error when evaluating patients diagnosed with this post operatively. This limits the potential patient pool and preoperative data to compare measures, ability to identify the true prevalence of UVCP in patients post cardiac surgery, and attrition due to lack of follow up or inability to reach patients, and very small sample size with only 14 patients being used in this study.

In contrast, a retrospective case series across four tertiary care pediatric hospitals done by Truong et al. noted that only 35% of patients had spontaneous recovery of VCP symptoms (17). This study identified 109 patients that were diagnosed with unspecified vocal cord paralysis after cardiac surgery, with the majority having undergone PDA ligation as at least part of their surgery. Patients included in the study had greater than three-month post operative follow up, with a mean time to spontaneous recovery defined by recovery of the vocal cord motion of 6.6 months. This study noted premature patients were more likely to have VCP post operatively and were less likely to have spontaneous recovery. Just under half (45%) of patients who also underwent an instrumental swallow evaluation showed aspiration or laryngeal penetration, and 27% of VCP patients required surgical intervention. This study faced the same limitation with respect to retrospectives design, providing limited data on post operative symptoms and diagnoses of VCP. This also does not account for patients with UVCP that were asymptomatic or resolved before requiring an otolaryngology consultation (14).

Similar to Truong's study, Orb et al. reported only 25% of patients had full resolution of symptoms and vocal cord movement, whereas 42% had resolution of symptoms by compensation of the contralateral vocal cord (19). The remaining patients (33%) were symptomatic at the time of follow up and required intervention (tracheostomy, injection medialization or laryngeal reinnervation).

Converse to these studies, De Gaudmar et al. conducted a retrospective review of pediatric patients with congenital VCP and excluded iatrogenic VCP, reporting 89% of patients to have spontaneously recovered based on an endoscopic evaluation using FNL, and oesophagoscopy (33). This study found most patients spontaneously recovered before 6 months of age and advocated waiting at least one year to allow for spontaneous recovery before offering surgical options; the recommendation to wait at least one year for spontaneous recovery has been suggested in other studies, such as Troung et al. (17).

Though this study reported a higher rate of spontaneous recovery than others, they excluded the iatrogenic population, which accounts for the largest population of UVCP patients as discussed above. This presents a major limitation regarding the generalizability of the study to the whole UVCP population. This study also conflated UVCP and BVCP, which is common across laryngeal paralysis literature. This is highly problematic due to the many differences in symptoms, etiologies, prognosis, and treatments for these two conditions as previously described.

Standardized follow up protocols and follow up data for patients with UVCP are also varied across the literature. For example, Orb et al. conducted a study assessing flexible laryngoscopy findings on neonates that underwent PDA ligation (19). Time of follow-up varied with the average follow up timing at 23 months post operatively. Cohen et al. also noted in their retrospective study that there are limited data from UVCP patients as they are often lost to follow up (14). Attrition of participants in UVCP studies due to resolution of symptoms and inconsistent follow up times (15) limit the follow up data in this patient population.

Though these studies provide some insight into the natural history of UVCP, they lack consistency in patient pool, objective measures of symptoms and were all retrospectively evaluating the natural history of the condition. The rate of spontaneous recovery ranged from 35% to 89% (17, 18, 33), which can make it difficult to estimate the true rate of spontaneous resolution.

Some studies suggest there are two groups associated with spontaneous recovery: an early recovery group where symptoms resolve within one year of onset, and a late group where symptoms recover longer than 18 months after the nerve has been damaged (29). A retrospective longitudinal cohort study by Prestwood et al. identified these two groupings, hypothesizing the severity of nerve damage will impact whether a patient will recover spontaneously in the early group, late group, or not at all (5). This information can assist surgeons with deciding which treatment option might be best for the patient—whether a watch and wait approach is recommended to allow for spontaneous recovery, or if surgical intervention may be offered if spontaneous resolution is unlikely. More prospective research needs to be done to test this theory.

In summary there is evidence of spontaneous recovery of vocal cord function in patients with UVCP; however, the main limitation of this claim is the assumption of recovery based on lack of follow up information, and the varying definition of recovery in the literature (symptom improvement vs. vocal cord motion recovery). What is particularly problematic for clinicians is how to determine the appropriate timeframe to allow for spontaneous recovery before recommending surgical intervention, as prolonged time to intervention has been reported to have worse long-term outcomes in both children and adults (55). Surgical intervention for UVCP has shown to have positive outcomes (6, 20, 25, 26, 56, 57); the application of these surgical techniques in children needs further evaluation to determine the best treatment recommendations for this population. The literature includes both perspectives, where some studies advocate for allowing time for spontaneous resolution of symptoms in 60% of cases (31) and recognize symptoms may improve without regaining movement of the vocal cord, whereas other studies report the benefits of earlier surgical intervention (4, 6, 17, 24, 31).

### Surgical methods

Some patients who have not had spontaneous resolution of UVCP and have persistent symptoms may be candidates for surgical intervention. Thyroplasty, IM, and more recently, RLNR have all been described as surgical interventions for patients with UVCP (please refer to Table 3). While thyroplasty is more commonly performed in adults (11), injection medialization (also referred to as injection laryngoplasty) and RLNR have both shown positive short-term outcomes in voice and swallowing symptoms (20). There is evidence that RLNR can improve voice and swallowing outcomes in pediatric patients (11, 22, 23, 25, 58), and injection laryngoplasty's limitations have given otolaryngologists reason to recommend RLNR as a more viable option (3, 22). Follow-up to evaluate the long-term outcomes of this patient population treated both surgically and non-surgically is not consistent across studies, which has proven to be a limitation in various reviews of the literature (4, 13, 26, 27).

**Table 3:** Summary of surgical interventions for UVCP.

Surgical intervention	Anesthetic	Indication for procedure	Time before symptom improvement	Need for repeat procedure
Injection Medialization	General, local (in adults)	• Young age	Immediately	Will need repeat procedure ∼every 6 months
		• Chance of spontaneous resolution (symptomatic <1-year)		
		• Aspiration risk/voice concerns		
Thyroplasty	Local or general	• Aspiration risk	Immediately	Potential need for repeat procedure
		• Adults/older children		
Recurrent Laryngeal Nerve Reinnervation	General	• Unlikely to spontaneously resolve (symptomatic >1-year)	3–6 months	No need for repeat procedure
		• Voice concerns		

#### Thyroplasty

Thyroplasty has been shown to have positive voice and swallowing outcomes on patients, though the literature has mostly focused on the adult population. It was initially developed by Ishikki et al., altering the thyroid cartilage and positioning of vocal cords to improve glottic closure (59). Ishikki presented four surgical techniques to perform the procedure: Type I medialized the paralyzed vocal cord, Type II lateralized the vocal cord, Type III shortened the vocal cords and Type IV lengthened the vocal cord by moving it anteroposteriorly (59).

Type I thyroplasty is more often used for UVCP treatment to medialize the paralyzed or atrophic vocal cord and allow for sufficient glottic closure with the healthy vocal cord (59). This procedure may be offered to patients with partial nerve input from the RLN or movement of the vocal cord, neurological contraindications for reinnervation surgery, or larger posterior glottic gap. Though most of the literature on medialization thyroplasty procedures is published on the adult population, there are studies identifying potential opportunities and indications for thyroplasty procedures to be performed on children (60–62).

Thyroplasty has been a viable option for many patients with UVCP, though it does have some limitations that have stimulated some surgeons to look for more feasible options in the pediatric space. A study in Cincinnati reported outcomes of the first cohort of pediatric patients to undergo a thyroplasty procedure (63). The surgeons performed the procedure with the same anatomical landmarks for adult patients on the pediatric group and found this did not result in ideal placement for the prosthetic. This study found the pediatric larynx was more challenging to insert the prosthesis due to the smaller anatomical area, and the more inferior location of the vocal fold. This resulted in the need for modification of the thyroplasty type I procedure for the pediatric population to account for the anatomical differences in laryngeal anatomy with age (63).

A recent retrospective study by Fayoux et al. that evaluated patients under 10 years of age who underwent Thyroplasty type 1 procedures reported positive longitudinal outcomes (one year post operative evaluation at minimum) (60). This study included patients between 8 months and 9 years of age, with aspiration and bronchopulmonary congestion as the main presenting complaints. Post operative evaluations by parents and providers showed improvements in aspiration, bronchopulmonary infections and voice quality based on the GRBAS scale. This study used a novel surgical technique, implanting autologous cartilage rather than the standard Gortex or Silastic implants. A similar study published by Gardener et al. reviewed two pediatric patients who underwent thyroplasty type 1 procedures, both with positive voice outcomes post operatively (61). This study also highlighted challenges performing this procedure on children, noting the difference in anatomy, limited feasibility to perform the operation on the awake child, and the potential effects of laryngeal framework procedures on the child's growing larynx (61).

A study by Wolter et al. analyzed surgical outcomes for medialization laryngoplasty in their centre, however they combined post operative outcomes for patients who had any medialization procedure, including injection laryngoplasty along with medialization thyroplasty (62). This study reported positive outcomes for voice and swallowing function by patient and parent perceptual reports for both procedures, with thyroplasty presenting with higher perceptual positive outcomes with regard to swallowing function (62).

A major limitation for the thyroplasty procedure is the possibility for revision surgeries as the larynx grows and changes with age, particularly for young children who have not reached puberty. Fayoux et al. experimented with autologous cartilage implants hypothesizing this would decrease the chances of requiring revision surgery, however more longitudinal research is needed to confirm this (60). Thyroplasty is often done on awake patients to test phonation and potentially visualize the movement of the vocal cords while adjusting the shape and size of the prosthetic. For these reasons, it is not commonly performed on pediatric patients. Studies have shown that although voice outcomes can be positive, there are limitations that do not allow the patient to use their voice in a higher pitch or for longer periods of time (64).

In summary, thyroplasty has had positive subjective outcomes for aspiration and voice symptoms. For specific post-pubescent patients whose larynx will not grow or change size, thyroplasty may serve as a reasonable option. Differences in adult and child laryngeal anatomy, need for revision surgery, and varied post operative results serve as limitations to this procedure in children. More research is needed to identify the appropriate pediatric patient population for this procedure, necessary adjustments in surgical techniques, and long-term objective outcomes after medialization thyroplasty in children.

#### Injection medialization

Injection medialization (IM) is the most common procedure offered to children with UVCP (65). IM involves the injection of an absorbable filler material lateral to the thyroarytenoid muscle causing the vocal cord to rest in a more medial position. This allows for glottic closure and has been shown to improve aspiration and voice symptoms (66, 67). This technique is used to alleviate symptoms while allowing for spontaneous recovery before opting for more invasive surgical procedures such as thyroplasty or laryngeal reinnervation. This procedure can be used in conjunction with other procedures, specifically RLNR.

IM was introduced by Bruening, who originally used Paraffin material as a treatment for UVCP (57). This was found to cause adverse reactions and was eventually replaced with Teflon (polytetrafluoroethylene) injections. This material was widely used until the 1990s after which it was found to have adverse long-term effects, like that of the paraffin injections (57). Since then, surgeons have experimented with many materials including carboxymethylcellulose, autologous fat, bovine collagen, cadaveric dermis, hyaluronic acid, or gel implants such as hydrated porcine gelatin powder or calcium hydroxyapatite. These have been found to have positive short-term outcomes, but long-term data is limited, especially in the pediatric population.

According to a recent study interviewing current American Society of Pediatric Otolaryngology (ASPO) members, hydroxy methylcellulose was used most often for IM, and aspiration was the most common indication for this surgery in infants (68).

In Aires et al.'s meta-analysis on surgical interventions (20), they found aspiration to be the main indication for IM, with carboxymethylcellulose being the most used injection material, supporting Jang et al.'s study (68), with about a 10% complication rate. In contrast, Butisky et al. found dysphonia to be the main indication for IM and cadaveric dermis to be the most common injectable material (6). Both studies demonstrated positive subjective outcomes but highlighted the lack of objective short- and long-term outcomes on voice and swallow function (6, 20).

One centre published a retrospective review of patients that had IM with either carboxymethylcellulose (Radiesse® Voice/Prolaryn® Plus, or Radiesse®/Prolaryn® voice Gel) or cadaveric dermis (Cymetra™) and found similarly positive results between groups. Time needed before a repeat injection did not vary based on primary symptoms, etiologies, or injection material (69).

Ayoub et al. recently published a study analyzing infants under 1 year of age who underwent IM (49). IM was performed with hyaluronic acid or carboxymethylcellulose in infants with positive aspiration on FEES assessments. A lower number of patients presented with penetration or aspiration, and an increased number of patients advanced their diet consistency. Results showed no negative outcomes or side effects in this patient group. Cates et al. conducted a study comparing a validated swallow study measure (EAT 10 survey) before and after either IM or thyroplasty (70). Approximately 20% of patients reported having normal swallowing scores post operatively which did not vary based on surgical procedure.

The limitations of IM include the transient nature of the dissolvable material and unknown long-term outcomes. Hartyl et al. published two cases that underwent autologous fat injection in adults with positive voice outcomes, however symptoms returned one month post operatively (66). Sipp et al. found similar results using cadaveric dermis, autologous fat, bovine collagen, hydrated porcine gelatin powder, and calcium hydroxyapatite, reporting utility for a maximum of 6 months (65). Injection medialization also may not aid in the glottic closure of patients with large posterior glottic gaps (57), due to the anatomical restrictions of the vocal ligament. This is one limitation of IM as a surgical intervention for children with UVCP.

In summary, IM has shown positive outcomes in the literature, but it lacks consistency on which materials are best used in children and does not show consistent objective outcomes proven with validated measurements. The transiency of this surgical technique is a limitation for the pediatric population, as they would require repeated sedated procedures. Though this is a viable option for patients with less severe symptoms or those looking for symptom improvement while waiting for spontaneous resolution, it lacks long-term efficacy.

#### Laryngeal reinnervation

Laryngeal reinnervation surgery has become increasingly popular as a surgical intervention for UVCP, but there is still limited data on the appropriate age for intervention and long-term outcomes in the pediatric population. Laryngeal reinnervation surgery can be recommended as a long-term treatment option for patients with UVCP that do not have any damage to the vagus nerve. There are three main surgical techniques reported to perform ansa-cervicalis recurrent laryngeal nerve innervation: Nerve-muscle pedicle transfer, nerve implantation and nerve suture (6). These surgical techniques can be further divided into selective or non-selective laryngeal reinnervation; selective laryngeal reinnervation anastomoses the ansa cervicalis to a specific recurrent laryngeal nerve branch that is responsible for innervating either an abductor or adductor muscle of the vocal cords. Non-selective laryngeal reinnervation anastomoses the ansa cervicalis to the main branch of the recurrent laryngeal nerve, innervating both abductor and adductor muscles.

A recent systematic review by Hoey et al. studied patient demographics and outcomes of pediatric patients who underwent laryngeal reinnervation (26). 19 articles met inclusion criteria with a total of 179 patients. There was a lack of data noting the age of injury or duration of paralysis, however the mean age for reinnervation was 8.6 years. They found iatrogenic injury to be the most common cause of the condition, with PDA ligation being the most common surgery. There were heterogenous protocols for assessment and documentation to confirm paralysis preoperatively with 25% of studies having no documentation of laryngeal assessment. There was limited use of EMG recordings, which was only documented in about 40% of studies. They found the age of injury and presentation to otolaryngology was inconsistent. Flexible nasal laryngoscopy was most often used, and EMG was used less commonly to examine and diagnose vocal cord paralysis.

This study reviewed reported surgical techniques and measured post operative outcomes with regards to voice, swallowing and quality of life measures for patients with UVCP and BVCP. There were differences in surgical technique in the placement of the initial incision and lack of specificity in which ansa cervicalis branch was used for reinnervation, with only 25% of studies reporting these details. There were also inconsistencies in the use of IM either prior to or at the same time as the innervation procedure. Post operative laryngeal assessments for UVCP have extremely limited documentation, with less than half of the studies documenting glottic closure or vocal fold movement post operatively. The authors note that most papers report subjective voice outcomes, using parent evaluation or surveys such as PVRQOL, or clinical evaluations such as the GRBAS ratings. MPT recordings were the most common objective voice evaluations across studies. Swallowing symptoms are less frequently reported, with parent evaluation being most documented.

Low numbers are a consistent issue, with over half of the included studies having less than 4 patients and only 4 studies having a “large” patient number of over 20 patients. This review also conflated BVCP with UVCP, which is another consistent limitation in the literature as noted previously. The lack of agreement on the protocol to diagnose vocal cord paralysis, as well as a clear definition of the condition is also lacking across studies. The authors noted most papers reporting retrospective evaluations of laryngeal reinnervation, lack of comparison between IM and RLNR outcomes, and heterogeny of voice and swallowing outcome measures are the main limitations of the available literature.

Tucker et al. were the first group to publish a case series on the nerve muscle pedicle (NMP) reinnervation technique on humans in the 1970s (71). Their goal was to improve the voice while maintaining airway to avoid tracheostomy in patients with BVCP. The NMP technique involves dissecting a branch of the ansa cervicalis muscle along with a small block of the muscle it innervates; in this study the branch of the ansa cervicalis inserting into the anterior belly of the omohyoid muscle was used. The nerve and muscle block are then implanted into the posterior cricoarytenoid muscle to allow for spontaneous abduction of the vocal cord. This group published a study reporting seven patients that had undergone this surgical technique, showing results for five patients with a mean follow up ranging between 5 months and 2 years. Patients underwent suspension laryngoscopy with laryngeal EMG prior to the reinnervation surgery to confirm absence of neural input to the posterior cricoarytenoid muscle. They found positive post operative outcomes with spontaneous abduction of the posterior cricoarytenoid muscle during inspiration on direct laryngoscopy on all five patients included in the study. No objective voice or swallow measures were noted; however, Tucker did report decannulation of three patients post operatively. Limitations of this study include the low patient number (5), variation in age, difference in duration of vocal cord paralysis, and lack of objective voice and swallow outcomes. While this study only included patients with BVCP, it is still a relevant surgical technique for this condition. More research is needed to understand the utility of the NMP technique for UVCP patients.

Fayoux et al. evaluated the utility of this surgical technique in pediatric patients in 2020 (72). This was a retrospective case series evaluating patients who had both bilateral and unilateral vocal cord paralysis in the adducted position who underwent the NMP reinnervation technique. Patients underwent suspension laryngoscopy with palpation of the vocal cords to rule out ankylosis and EMG recordings were taken. Dyspnea and voice quality were measured pre and post operatively, and vocal cord abduction was categorized into four groups as outlined by the authors. Fayoux noted success in the NMP procedure with spontaneous abduction of the previously paralyzed vocal cord in most patients during a post operative FNL evaluation within 6 months of the surgery (72). With long-term follow up, they found about half of the patients had resolution of dyspnea symptoms with a lower rate of success in voice outcomes based on the GRBAS scale. The authors did not use objective measurements to evaluate post operative outcomes and did not define the variables they were analyzing, which are important limitations. Many patients in this cohort had concomitant airway disease and previous surgeries, which make it difficult to pool patient treatments and outcomes together. This study also combined BVCP and UVCP patients, which as noted previously are not comparable due to the differences in etiologies, prognosis, and general treatment options.

The NMP surgical technique has limited results in the literature, and further research is needed to determine the objective voice, swallow and respiratory outcomes of patients who undergo this procedure.

Nerve implantation reinnervation has been studied mainly in adults (73, 74). This surgical technique involves the insertion of the ansa cervicalis nerve into the thyroarytenoid muscle; this differs from the NMP technique as it implants the nerve into the thyroarytenoid muscle rather than the posterior cricoarytenoid muscle. Authors claim that because this technique does not require locating the denervated or damaged RLN, it is advantageous in its simplicity and shorter procedure time compared to other reinnervation procedures. The Su et al. series reported on 10 patients with varied etiologies including cardiac surgery, thyroidectomy or parathyroidectomy, idiopathic and spinal cord procedures diagnosed with UVCP were selected to receive the nerve implantation procedure. Preoperative assessments included video laryngoscopy, GRBAS ratings, perturbation measures and maximum phonation times were collected. Some patients also had pre and post operative laryngeal electromyography (LEMG) recordings documented. Post operative outcomes showed some, but not all patients had improved voice outcomes post operatively, which the authors suspect was due to unsuccessful reinnervation of the TA muscle in some patients. The authors reported improved positioning of the vocal cords and arytenoids in most of the patients, described as symmetrical with phonation. Careful selection of the appropriate candidate for this surgery is necessary; the authors concluded that this technique is most useful for patients with unusable RLN stumps and require reinnervation of the thyroarytenoid muscle for vocal cord adduction. More research is needed to evaluate if this technique is a viable option for the pediatric population.

The most reported laryngeal reinnervation surgical technique is the nerve suture technique, anastomosing the ansa cervicalis and recurrent laryngeal nerve to improve muscle bulk and tone to the paralyzed vocal cord. This allows functional adduction and phonation by the compensatory movement of the non-paralyzed vocal cord. The nerve suture technique was originally published by Frazier et al. and later practiced by Crumley (22, 23), presenting a case series of two patients who underwent this procedure (22). Patients presented with dysphonia as the primary symptom and voice quality was the main outcome measured post operatively. Crumley reported this surgical technique leads to improvement of symptoms and advocated to continue its use with more patients.

A case series discussing the non-selective laryngeal reinnervation surgical technique in children was published in 2020, noting key points that may help surgeons have a successful reinnervation and tension-free anastomosis (58). This article collected data from 21 patients between the ages of 3 and 14 years old who had UVCP from varied etiologies including thoracic surgery, esophageal surgery, neurologic surgery, laryngotracheal reconstruction and idiopathic. Patients were originally seen for aspiration or voice symptoms. Improvement of voice symptoms was characterized by subjective assessment from the provider, caregiver, and patient. The authors defined resolution of aspiration symptoms by the ability to change feeding method by at least one half-consistency. With relation to surgical technique, this article highlighted the importance of ansa-cervicalis identification, understanding the variants of anatomy in relation to the carotid sheath to preserve the nerve integrity and protect surrounding structures. All patients underwent injection medialization prior to and during RLNR; the authors did not specify if they used selective or non-selective RLNR technique, however they did note the RLN was dissected “with about 1–2 cm of length to cricothyroid joint” (58). Patients were followed post operatively between 6 months to 16 months to evaluate voice and swallow symptoms. The authors reported positive voice and swallowing outcomes for almost all patients, with 19 of the 21 patients reporting resolution of voice or swallowing symptoms. Two patients who presented with both voice and swallow symptoms reported only having one symptom resolve post operatively. Overall, the authors supported this surgical approach and advocated for ansa-RLN reinnervation as a viable option for UVCP in children.

This case series identified surgical techniques that may assist in positive outcomes for RLNR surgery, including a tension-free anastomosis, understanding of anatomical variations, and neurorrhaphy repair rather than nerve-muscle pedicle repair. This study showed positive post operative results, however there were some limitations to the study that should be noted. The study was done at a single centre, with a limited number of only 21 patients. There were no reports of diagnostic assessments pre or post operatively to confirm the presence and resolution of UVCP. There were no objective measures taken to analyze voice quality pre and post operatively. The evaluation of aspiration symptom resolution was determined by increasing the thickness of consistency, but no clinical or instrumental swallow assessments were reported. Overall, this study was helpful in describing the RLNR surgical technique that may improve outcomes in children, however more objective evaluations of symptoms is needed to understand the impact of this surgery on UVCP.

Ongkasuwan et al. published a study evaluating voice outcomes of children that underwent non-selective recurrent laryngeal nerve reinnervation with follow up after one year (24). Patients underwent pre and post operative laryngoscopies to diagnose and evaluate UVCP, having EMG recordings to confirm the absence of neural input to the internal laryngeal muscles. Patients received injection medialization at the time of the EMG procedure, before the RLNR surgery. Objective voice measures were taken using two validated voice measurements, PVRQOL survey and CAPE-V to evaluate voice quality pre and post operatively. Other validated objective measures of voice including MPT, jitter, shimmer and noise to harmonic ratio were also taken. All objective measures were analyzed by registered speech language pathologists and ADSV software. Voice measures were taken at 4,6-,9- and 12-month intervals post operatively. Analysis of objective voice measures showed improvement in 10 of the 14 measures analyzed. Posterior glottic gap resulted in lower PVRQOL scores post operatively. The authors concluded non-selective reinnervation results in positive outcomes, with none of their patients experiencing a decline in voice quality post operatively. They also found older patients had higher MPT pre and post operatively, but longer duration of UVCP did have lower post operative MPTs suggesting earlier intervention may result in better voice quality.

Similar to Caloway's study (58), the low patient number and fact that this study was done at a single institution are limitations to this study (24). The study did not clarify when patients had IM before RLNR, which could potentially impact the results during follow up voice evaluations if the injection was still present. Another limitation of this study is the attrition of participants, with 1/3 of patients lost to follow up, losing some of the potential data. This study showed non-selective RLNR can positively impact voice quality in children, but replication of this data, evaluation of other UVCP symptoms and long-term follow up is still needed.

Zur et al. published a case series evaluating the utility of RLNR surgery as a management option for aspiration in children (25). This study described three patients aged 5, 6 and 10 who underwent cardiac surgery and were diagnosed with UVCP (with one patient also presenting with synkinetic movement of the left vocal and arytenoid folds). Each patient presented with aspiration of liquids based on instrumental swallow studies preoperatively and confirmation of UVCP with otolaryngologist evaluation and EMG study. Patients underwent RLNR with either the omohyoid branch or the sternothyroid branch of the ansa cervicalis. Some voice measurements were taken pre and post operatively, including GBRAS ratings, VHI, MPT, perturbation and vocal range. The authors suggest the RLNR procedure assists with glottic closure and protection of the lower airway to mitigate aspiration symptoms and complications.

This case study provided insight into possible utilization of RLNR in children presenting with chronic or recurrent aspiration, however it is difficult to base conclusions or generalize these concepts due to the low patient number, single institution, variation in surgical technique between patients, non-specificity of pre and post op evaluations, variation in patient history, and lack of long term follow up.

When comparing surgical interventions for UVCP, RLNR seems to consistently show positive outcomes, though the amount of published research and long-term data is sparce. Zur et al. have compared post operative outcomes of RLNR with injection laryngoplasty (25). This was a retrospective chart review of 33 children who presented with dysphonia and UVCP as the main diagnosis, with varying etiologies including cardiac surgery, tumor excision or other airway procedures. Participants underwent a laryngeal evaluation by flexible endoscopy and were divided into three groups based on the treatment option they decided to pursue; they were given the option to observe symptoms without surgical intervention, undergo IM or RLNR procedures. Laryngeal imaging was done using stroboscopy and analysis of acoustic measures was done using VisiPitch software. This study compared voice capacity ratings with dysphonia scales in pediatric patients with UVCP and compared voice outcomes between the three treatment groups (25).

This study found that patients with cardiac surgery history were less likely to choose RLNR surgery, whereas extremely premature patients were more likely to choose RLNR (21). Premature patients were found to have more severe dysphonia than full-term patients. RLNR patients had better outcomes when comparing post op VHI ratings compared to IM. They also found that non-surgical patients had worse GRBAS scores than post operative surgical patients. RLNR outcomes did not significantly differ based on duration of UVCP preoperatively. They recommended a treatment algorithm for physicians to follow with UVCP, recommending voice therapy for all patients regardless of their decision on surgery, and have injection laryngoplasty as a short-term option or for patients that still have positive neural response findings on their EMG. For patients with no RLN output on their EMG and who need a long-term solution, RLNR is recommended in comparison to injection medialization (25). The limitations of this study included its retrospective design, low patient numbers and that patients selected their treatment group, which may have resulted in bias in the results.

A study by Paniello et al. compared medialization laryngoplasty with recurrent laryngeal nerve reinnervation surgeries in adults (75). They found patients that had the medialization procedure had better results than RLNR, based on expert and non-expert acoustic ratings, PVRQOL survey results and MPT. The group that had ML had better results after a 6-month period, and both groups had equal outcomes after 12 months. Neither group had “perfect” or “normal” voice ratings after the procedure. This study concluded that ML might be better for the “older” group (above 52 years of age). One reason could be that nerve regeneration and adaptation is better and more likely in younger patients (75).

One major limitation of RLNR is the time needed to assess if reinnervation was successful and use of injection medialization in conjunction with laryngeal reinnervation surgery, consistently noted in the literature (20, 21, 24). Because nerve reinnervation can take up to 6 months to show positive outcomes, surgeons often use injection medialization at the time of the reinnervation to accelerate recovery times. For some cases, this can result in uncertainty if the reinnervation, injection medialization or spontaneous resolution of paralysis is responsible for the resolution of symptoms. This is why many studies opt to wait for at least 12 months to allow for spontaneous resolution before offering surgical intervention (20).

#### Surgical outcomes

While each surgical technique has been reported to have positive outcomes in pediatric patients, there is still discussion surrounding ideal patient groups for each intervention, best timing for intervention and lack of prospective and long-term data.

Butisky et al. published a systematic review reporting 15 studies with only 84 patients that described surgical intervention for UVCP as of 2015 (6). These studies included six observational studies, six case series and three case reports. Six studies reported outcomes of injection laryngoplasty, five case reports documented outcomes of thyroplasty, and eight studies documented outcomes after laryngeal reinnervation. Dysphonia was the most common indication for injection laryngoplasty, though this procedure showed positive outcomes for both voice and swallowing. Thyroplasty was used on patients that presented with dysphonia and aspiration, however there were no objective evaluations of voice in any of the studies. This procedure showed more positive outcomes in swallowing symptoms (88%) compared to voice (42%). The studies evaluating RLNR were only case series and case reports which serves as a major limitation in this area due to the high risk of bias (Level of evidence = 5). Dysphonia was present in all but one of the patients who underwent RLNR, making this the most common indication for this procedure. The authors note most studies only reported post operative subjective and objective measures of voice, while follow up times and measurements varied across studies. All studies reported improvement in voice symptoms and time for symptom improvement was between 3 and 7 months.

These authors suggested that injection medialization is a safe and effective temporary treatment for UVCP, whereas thyroplasty and reinnervation techniques should be offered as permanent options for patients who are unlikely to have spontaneous recovery. This study noted the lack of long-term follow up for these surgical procedures. There were also inconsistencies in the objective outcomes used to evaluate voice and swallowing after surgery (6). Limitations of this review included the lack of consistency around pre and post operative evaluations, low participant numbers, and lack of high-level evidence across the literature (maximum level 4 evidence).

Aires et al. published an updated systematic review and meta-analysis in 2020 (20). This review included 22 articles that focused on outcomes of injection laryngoplasty and laryngeal reinnervation. Seven articles on laryngeal reinnervation were used for a meta-analysis of the MPT and GRBAS measures. A total of 267 patients were documented with the highest participant number in one study being 41. Contradictory to Hoey et al.'s review (26), aspiration was the main indication for injection medialization rather than dysphonia. They also reported a complication rate of 11.5%, which was higher than previously reported. The authors found cardiac surgery, specifically PDA ligation were the most common etiologies for both IL and RLNR patients.

The authors found both injection laryngoplasty and laryngeal reinnervation show positive outcomes for both voice and swallowing based on MPT and GRBAS objective measures, however the meta-analysis concluded high publication bias and high heterogeneity index, with only retrospective studies or case studies meeting the inclusion criteria (20) This study concluded that IM is a viable temporary option for UVCP and RLNR is a promising long term treatment option for UVCP. The authors noted that the main limitation to this meta-analysis was the lack of prospective data with long term follow up and uniform outcome measurements. This study defined pediatric as ages 0–21, whereas other studies have used patients 0–18, which should be noted. Another limitation was the low number of studies used in the meta-analysis (only 3 for the analysis of GRBAS rating), which makes this data difficult to derive robust conclusions. This study is not generalizable due to the heterogeneity of measurements, follow ups, age at intervention and high risk of bias in the included studies.

## Conclusion

UVCP is a growing research area in pediatric medicine. It is known that iatrogenic injury is a common cause of this condition, due to the anatomical route of the recurrent laryngeal nerve and course along the cardiac structures. The true prevalence of this condition is not well documented in the literature due to varying definitions, limited documentation, and protocols for diagnosing vocal cord paralysis. Though there is a growing number of studies evaluating UVCP diagnosis and treatment in children, there is still more research needed to help standardize the diagnosis, understand the natural history, burden of swallowing, voice and airway symptoms, and long-term outcomes in pediatric patients that are treated both surgically and non-surgically. Novel surgical therapies require validation in order to expand their use.

## References

[1] Chhetri DK Berke GS. Ansa cervicalis nerve: review of the topographic anatomy and morphology. Laryngoscope. (1997) 107(10):1366–72. 10.1097/00005537-199710000-00014 9331315

[2] Kruse E Olthoff A Schiel R. Functional anatomy of the recurrent and superior laryngeal nerve. Langenbecks Arch Surg. (2006) 391(1):4–8. 10.1007/s00423-005-0011-7 16374605

[3] Daya H Hosni A Bejar-Solar I Evans JN Bailey CM. Pediatric vocal fold paralysis a long-term retrospective study. Arch Otolaryngol Head Neck Surg. (2000) 126(1):21–5. 10.1001/archotol.126.1.21 10628706

[4] Jabbour J Martin T Beste D Robey T. Pediatric vocal fold immobility: natural history and the need for long-term follow-up. JAMA Otolaryngol Head Neck Surg. (2014) 140(5):428–33. 10.1001/jamaoto.2014.81 24626342

[5] Prestwood CA Brown AF Johnson RF. Recovery of vocal cord motion among pediatric patients. Ann Otol Rhinol Laryngol. (2022) 131(6):587–94. 10.1177/00034894211033366 34282639

[6] Butskiy O Mistry B Chadha NK. Surgical interventions for pediatric unilateral vocal cord paralysis: a systematic review. JAMA Otolaryngol Head Neck Surg. (2015) 141:654–60. 10.1001/jamaoto.2015.0680 25973887

[7] Jabbour J North LM Bougie D Robey T. Vocal fold immobility due to birth trauma: a systematic review and pooled analysis. Otolaryngol Head Neck Surg. (2017) 157(6):948–54. 10.1177/0194599817726773 28871836

[8] Sulica L. The natural history of idiopathic unilateral vocal fold paralysis: evidence and problems. Laryngoscope. (2008) 118(7):1303–7. 10.1097/MLG.0b013e31816f27ee 18496160

[9] Rosin DF Handler SD Potsic WP Wetmore RF Tom LWC. Vocal cord paralysis in children. Laryngoscope. (1990) 100(11):1174–9. 10.1288/00005537-199011000-00008 2233079

[10] Friedman EM. Role of ultrasound in the assessment of vocal cord function in infants and children. Ann Otol Rhinol Laryngol. (1997) 106(3):199–209. 10.1177/000348949710600304 9078931

[11] Graham ME Smith ME. Unilateral vocal fold immobility in children. Otolaryngol Clin North Am. (2019) 52(4):681–92. 10.1016/j.otc.2019.03.012 31072641

[12] Kashima K Watanabe K Sato T Katori Y. Analysis of dysphagia and cough strength in patients with unilateral vocal fold paralysis. Dysphagia. (2023) 38(2):510–6. 10.1007/s00455-021-10274-8 33728514

[13] Strychowsky JE Rukholm G Gupta MK Reid D. Unilateral vocal fold paralysis after congenital cardiothoracic surgery: a meta-analysis. Pediatrics. (2014) 133(6):e1708-23. 10.1542/peds.2013-3939 24843065

[14] Cohen SR Geller KA Birns JW Thompson JW. Laryngeal paralysis in children: a long-term retrospective study. Ann Otol Rhinol Laryngol. (1982) 91(4 Pt 1):417–24. 10.1177/000348948209100420 7114725

[15] Orzell S Joseph R Ongkasuwan J Bedwell J Shin J Raol N. Outcomes of vocal fold motion impairment and dysphagia after pediatric cardiothoracic surgery: a systematic review. Otolaryngol Head Neck Surg. (2019) 161(5):754–63. 10.1177/0194599819858594 31234735

[16] Henry BM Hsieh WC Sanna B Vikse J Taterra D Tomaszewski KA. Incidence, risk factors, and comorbidities of vocal cord paralysis after surgical closure of a patent ductus arteriosus: a meta-analysis. Pediatr Cardiol. (2019) 40(1):116–25. 10.1007/s00246-018-1967-8 30167748 PMC6348263

[17] Truong MT Messner AH Kerschner JE Scholes M Wong-Dominguez J Milczuk HA Pediatric vocal fold paralysis after cardiac surgery: rate of recovery and sequelae. Otolaryngol Head Neck Surg. (2007) 137(5):780–4. 10.1016/j.otohns.2007.07.028 17967646

[18] Biot T Fieux M Henaine R Truy E Coudert A Ayari-Khalfallah S. Long term outcome of laryngeal mobility disorder and quality of life after pediatric cardiac surgery. Int J Pediatr Otorhinolaryngol. (2022) 158:111142. 10.1016/j.ijporl.2022.111142 35580383

[19] Orb Q Dunya G Padia R King J Holbrook J Muntz H Long-term outcomes of vocal fold paralysis following patent ductus arteriosus ligation in neonates. Laryngoscope. (2023) 133(5):1257–61. 10.1002/lary.30343 36054344

[20] Aires MM Marinho CB de Vasconcelos SJ. Surgical interventions for pediatric unilateral vocal fold paralysis: a systematic review and meta-analysis. Int J Pediatr Otorhinolaryngol. (2021) 141. 10.1016/j.ijporl.2020.110553 33333340

[21] Zur KB Carroll LM. Recurrent laryngeal nerve reinnervation in children: acoustic and endoscopic characteristics pre-intervention and post-intervention. A comparison of treatment options. Laryngoscope. (2015) 125:S1–15. 10.1002/lary.25538 26257068

[22] Crumley RL Izdebski K. Voice quality following laryngeal reinnervation by ansa hypoglossi transfer. Laryngoscope. (1986) 96(6):611–6. 10.1288/00005537-198606000-00004 3713403

[23] Crumley RL. Update: ansa cervicalis to recurrent laryngeal nerve anastomosis for unilateral laryngeal paralysis. Laryngoscope. (1991) 101(4 I):384–8. 10.1002/lary.1991.101.4.384 1895854

[24] Ongkasuwan J Espinosa MCL Hollas S Devore D Procter T Bassett E Predictors of voice outcome in pediatric non-selective laryngeal reinnervation. Laryngoscope. (2020) 130(6):1525–31. 10.1002/lary.28282 31498453

[25] Zur KB Carroll LM. Recurrent laryngeal nerve reinnervation for management of aspiration in a subset of children. Int J Pediatr Otorhinolaryngol. (2018) 104(October 2017):104–7. 10.1016/j.ijporl.2017.11.002 29287848

[26] Hoey AW Hall A Butler C Frauenfelder C Wyatt M. Laryngeal reinnervation for paediatric vocal cord palsy: a systematic review. Eur Arch Oto-Rhino-Laryngol. (2022) 279(12):5771–81. 10.1007/s00405-022-07471-y 35838782

[27] Francis DO Sherman AE Hovis KL Bonnet K Schlundt D Garrett CG Life experience of patients with unilateral vocal fold paralysis. JAMA Otolaryngol Head Neck Surg. (2018) 144(5):433–9. 10.1001/jamaoto.2018.0067 29621392 PMC6136051

[28] Kakodkar KA Schroeder JW Holinger LD. Laryngeal Development and Anatomy The Larynx (2012). Vol. 73. Available online at: http://karger.com/books/book/chapter-pdf/4037039/000334108.pdf (Accessed February 10, 2024). 10.1159/000334108 22472221

[29] Myssiorek D. Recurrent laryngeal nerve paralysis: anatomy and etiology. Otolaryngol Clin North Am. (2004) 37(1):25–44. 10.1016/S0030-6665(03)00172-5 15062685

[30] Gentile RD Miller RH Woodson GE. Vocal cord paralysis in children 1 year of age and younger. Ann Otol Rhinol Laryngol. (1986) 95(6 Pt 1):622–5. 10.1177/000348948609500616 3789596

[31] Emery PJ Fearon B. Vocal cord palsy in pediatric practice: a review of 71 cases. Int J Pediatr Otorhinolaryngol. (1984) 8(2):147–54. 10.1016/S0165-5876(84)80063-4 6526582

[32] Engeseth MS Olsen NR Maeland S Halvorsen T Goode A Røksund OD. Left vocal cord paralysis after patent ductus arteriosus ligation: a systematic review. Paediatr Respir Rev. (2018) 27:74–85. 10.1016/j.prrv.2017.11.001 29336933

[33] De Gaudemar I Roudaire M François M Narcy P. Outcome of laryngeal paralysis in neonates: a long term retrospective study of 113 cases. Int J Pediatr Otorhinolaryngol. (1996) 34(1–2):101–10. 10.1016/0165-5876(95)01262-1 8770677

[34] Dewan K Cephus C Owczarzak V Ocampo E. Incidence and implication of vocal fold paresis following neonatal cardiac surgery. Laryngoscope. (2012) 122(12):2781–5. 10.1002/lary.23575 22952115

[35] Spector BC Netterville JL Billante C Clary J Reinisch L Smith TL. Quality-of-life assessment in patients with unilateral vocal cord paralysis. Otolaryngol Head Neck Surg. (2001) 125(3):176–82. 10.1067/mhn.2001.117714 11555751

[36] Tibbetts KM Wu D Hsu JV Burton WB Nassar M Tan M. Etiology and long-term functional swallow outcomes in pediatric unilateral vocal fold immobility. Int J Pediatr Otorhinolaryngol. (2016) 88:179–83. 10.1016/j.ijporl.2016.07.008 27497409

[37] Zur KB Douglas J Carroll LM. Intubation-related laryngeal deficiency and vocal fold immobility in pediatric premature patients. Laryngoscope. (2021) 131(11):2550–7. 10.1002/lary.29592 33956345

[38] Boseley ME Cunningham MJ Volk MS Hartnick CJ. Validation of the pediatric voice-related quality-of-life survey. Arch Otolaryngol Head Neck Surg. (2006) 132(7):717–20. 10.1001/ARCHOTOL.132.7.717 16847178

[39] Yağcıoğlu D Aydınlı FE Aslan G Kirazlı MÇ Köse A Doğan N Development, validation, and reliability of the teacher-reported pediatric voice handicap index. Lang Speech Hear Serv Sch. (2022) 53(1):69–87. 10.1044/2021_LSHSS-21-00033 34762816

[40] Kempster GB Gerratt BR Verdolini Abbott K Barkmeier-Kraemer J Hillman RE. Consensus auditory-perceptual evaluation of voice: development of a standardized clinical protocol. Am J Speech Lang Pathol. (2009) 18(2):124–32. 10.1044/1058-0360(2008/08-0017) 18930908

[41] Labaere A De Bodt M Van Nuffelen G. Construction of an anchor and training sample set for auditory-perceptual voice evaluation with the GRBAS-scale. J Voice. (2023). 10.1016/j.jvoice.2023.09.033. [Epub ahead of print]. 37981533

[42] Belafsky PC Mouadeb DA Rees CJ Pryor JC Postma GN Allen J Validity and reliability of the eating assessment tool (EAT-10). Ann Otol Rhinol Laryngol. (2008) 117(12):919–24. 10.1177/000348940811701210 19140539

[43] Baqays A Johannsen W Rashid M Jaffal H Hicks A Jeffery C Parent-reported outcome questionnaire for swallowing dysfunction in healthy infants and toddlers: construction and content validation. Otolaryngol Head Neck Surg. (2021) 165(1):197–205. 10.1177/0194599820970950 33287657

[44] Connor NP Cohen SB Theis SM Thibeault SL Heatley DG Bless DM. Attitudes of children with dysphonia. J Voice. (2008) 22(2):197–209. 10.1016/j.jvoice.2006.09.005 17512168

[45] Behlau M Madazio G Oliveira G. Functional dysphonia: strategies to improve patient outcomes. Patient Relat Outcome Meas. (2015) 6:243. 10.2147/prom.s68631 26664248 PMC4671799

[46] Mornet E Coulombeau B Fayoux P Marie JP Nicollas R Robert-Rochet D Assessment of chronic childhood dysphonia. Eur Ann Otorhinolaryngol Head Neck Dis. (2014) 131(5):309–12. 10.1016/j.anorl.2013.02.001 24986259

[47] Liu YCC McElwee T Musso M Rosenberg TL Ongkasuwan J. The reliability of flexible nasolaryngoscopy in the identification of vocal fold movement impairment in young infants. Int J Pediatr Otorhinolaryngol. (2017) 100:157–9. 10.1016/j.ijporl.2017.07.005 28802364

[48] Meister KD Johnson A Sidell DR. Injection laryngoplasty for children with unilateral vocal fold paralysis: procedural limitations and swallow outcomes. Otolaryngol Head Neck Surg. (2019) 160(3):540–5. 10.1177/0194599818813002 30453837

[49] Ayoub N Balakrishnan K Meister K Grimm D Johnson A Maida K Safety and effectiveness of vocal fold injection laryngoplasty in infants less than one year of age. Int J Pediatr Otorhinolaryngol. (2023) 168:111542. 10.1016/j.ijporl.2023.111542 37058865

[50] Sheen D Houser TK Olsson SE Dabbous H Kou YF Johnson RF Injection laryngoplasty for children with dysphagia after cardiac surgery. OTO Open. (2024) 8(2):e142. 10.1002/oto2.142 38689853 PMC11058695

[51] Heman-Ackah YD Mandel S Manon-Espaillat R Abaza MM Sataloff RT. Laryngeal electromyography. Otolaryngol Clin North Am. (2007) 40(5):1003–23. 10.1016/j.otc.2007.05.007 17765693

[52] Koch BM Milmoe G Grundfast KM. Vocal cord paralysis in children studied by monopolar electromyography. Pediatr Neurol. (1987) 3(5):288–93. 10.1016/0887-8994(87)90070-1 3508076

[53] Maturo SC Braun N Brown DJ Chong PST Kerschner JE Hartnick CJ. Intraoperative laryngeal electromyography in children with vocal fold immobility: results of a multicenter longitudinal study. Arch Otolaryngol Head Neck Surg. (2011) 137(12):1251–7. 10.1001/ARCHOTO.2011.184 22183907

[54] Horner C Chan T Yip C Parikh SR Johnson K Fridgen J Improving timeliness of vocal fold mechanical injury screening following Norwood or arch reconstruction: a quality improvement initiative at a single center. Pediatr Cardiol. (2023) 44(2):388–95. 10.1007/s00246-022-03064-y 36527473

[55] Smith ME Houtz DR. Outcomes of laryngeal reinnervation for unilateral vocal fold paralysis in children: associations with age and time since injury. Ann Otol Rhinol Laryngol. (2016) 125:433–8. 10.1177/0003489415615364 26553660

[56] Farhood Z Reusser NM Bender RW Thekdi AA Albright JT Edmonds JL. Pediatric recurrent laryngeal nerve reinnervation: a case series and analysis of post-operative outcomes. Int J Pediatr Otorhinolaryngol. (2015) 79(8):1320–3. 10.1016/j.ijporl.2015.06.001 26093531

[57] Kwon TK Buckmire R. Injection laryngoplasty for management of unilateral vocal fold paralysis. Curr Opin Otolaryngol Head Neck Surg. (2004) 12(6):538–42. 10.1097/01.MOO.0000144393.40874.98 15548914

[58] Caloway CL Bouhabel S Hartnick CJ. Lessons learned to aid the successful outcome of pediatric recurrent laryngeal nerve reinnervation. Int J Pediatr Otorhinolaryngol. (2020) 128:109742. 10.1016/j.ijporl.2019.109742 31677453

[59] Isshiki N Morita H Okamura H Hiramoto M. Thyroplasty as a new phonosurgical technique. Acta Otolaryngol. (1974) 78(1–6):451–7. 10.3109/00016487409126379 4451096

[60] Fayoux P Maltezeanu A Lemesre PE Broucqsault H. Evaluation of thyroplasty with cartilage implant in young children. Int J Pediatr Otorhinolaryngol. (2023) 167:111488. 10.1016/j.ijporl.2023.111488 36878165

[61] Gardner GM Altman JS Balakrishnan G. Pediatric Vocal Fold Medialization with Silastic Implant: Intraoperative Airway Management (2000). Vol. 52. Available online at: www.elsevier.com/locate/ijporl (Accessed February 20, 2024). 10.1016/s0165-5876(99)00288-8 10699238

[62] Wolter NE Ayele N Kawai K Hseu A Nuss R. Medialization laryngoplasty in pediatric patients with unilateral vocal fold immobility: a case series. Ann Otol Rhinol Laryngol. (2019) 128(2):145–51. 10.1177/0003489418814276 30450941

[63] Link DT Willging JP Rutter MJ Myer CM Liu JH Cotton RT. Pediatric type I thyroplasty: an evolving procedure. Ann Otol Rhinol Laryngol. (1999) 108(12):1105–10. 10.1177/000348949910801201 10605912

[64] Hogikyan ND Wodchis WP Terrell JE Bradford CR Esclamado RM. Voice-related quality of life (V-RQOL) following type I thyroplasty for unilateral vocal fold paralysis. J Voice. (2000) 14(3):378–86. 10.1016/S0892-1997(00)80083-1 11021505

[65] Sipp JA Kerschner JE Braune N Hartnick CJ. Vocal fold medialization in children: injection laryngoplasty, thyroplasty, or nerve reinnervation? Arch Otolaryngol Head Neck Surg. (2007) 133(8):767–71. 10.1001/ARCHOTOL.133.8.767 17709613

[66] Hartl DM Travagli JP Leboulleux S Baudin E Brasnu DF Schlumberger M. Clinical review: current concepts in the management of unilateral recurrent laryngeal nerve paralysis after thyroid surgery. J Clin Endocrinol Metab. (2005) 90(5):3084–8. 10.1210/jc.2004-2533 15728196

[67] Laccourreye O Paczona R Ageel M Hans S Brasu D Crevier-Buchman L. Intracordal autologous fat injection for as- piration after recurrent nerve paralysis. Eur Arch Otorhinolaryngol. (1999) 256:458–61. 10.1007/s004050050189 10552226

[68] Jang M Gregory S Jabbour J Robey TC Sulman C Chun R. Injection laryngoplasty in infants with unilateral vocal cord paralysis: a survey of ASPO members. Int J Pediatr Otorhinolaryngol. (2020) 128:109671. 10.1016/j.ijporl.2019.109671 31756694

[69] Bishop R Mousset M Althubaiti A Gerwig A Kern C Onwuka A Effect of injection laryngoplasty material on outcomes in pediatric vocal fold paralysis. Transl Pediatr. (2022) 11(7):1114–21. 10.21037/tp-21-361 35957995 PMC9360818

[70] Cates DJ Venkatesan NN Strong B Kuhn MA Belafsky PC. Effect of vocal fold medialization on dysphagia in patients with unilateral vocal fold immobility. Otolaryngol Head Neck Surg. (2016) 155:454–7. 10.1177/0194599816645765 27165683

[71] Tucker HM. Human laryngeal reinnervation. Laryngoscope. (1976) 86(6):769–79. 10.1288/00005537-197606000-00004 933669

[72] Fayoux P Maltezeanu A Broucqsault H Daniel SJ. Experience with laryngeal reinnervation using nerve-muscle pedicle in pediatric patients. Int J Pediatr Otorhinolaryngol. (2020) 138:110254. 10.1016/j.ijporl.2020.110254 33137867

[73] Kodama N Sanuki T Kumai Y Yumoto E. Long-term vocal outcomes of refined nerve-muscle pedicle flap implantation combined with arytenoid adduction. Eur Arch Otorhinolaryngol. (2015) 272(3):681–8. 10.1007/S00405-014-3418-3 25502739

[74] Su WF Hsu YD Chen HC Sheng H. Laryngeal reinnervation by ansa cervicalis nerve implantation for unilateral vocal cord paralysis in humans. J Am Coll Surg. (2007) 204(1):64–72. 10.1016/J.JAMCOLLSURG.2006.08.028 17189114

[75] Paniello RC Edgar JD Kallogjeri D Piccirillo JF. Medialization versus reinnervation for unilateral vocal fold paralysis: a multicenter randomized clinical trial. Laryngoscope. (2011) 121(10):2172–9. 10.1002/lary.21754 21898419 PMC3183158

